# The Role of Exercise and Rehabilitation in the Cancer Care Plan

**DOI:** 10.6004/jadpro.2016.7.3.20

**Published:** 2016-04-01

**Authors:** Angelo Rizzo

**Affiliations:** Therapeutic Solutions, Inc, Oncology Rehabilitation and Lymphedema Clinic, Conyers, Georgia

The integration of an oncology physical therapist into the oncology team throughout the cancer survivor trajectory can benefit both advanced practitioners and survivors, according to Angelo Rizzo, MS, PT, CLT, President and Founder of Therapeutic Solutions, Inc, Oncology Rehabilitation and Lymphedema Clinic, Conyers, Georgia. Physical impairments of patients undergoing cancer treatment can impact functioning and quality of life throughout survivorship, but exercise and rehabilitation may mitigate some of these treatment-related adverse effects and comorbidities, he said.

"With advances in early detection and screening, and with improvements in treatment protocols, patients are living longer. Unfortunately, many are living with lifelong impairments as a result of their cancer treatment," Mr. Rizzo noted in his presentation at 2015 JADPRO Live at APSHO.

Cancer survivors are almost three times more likely to report fair or poor health after treatment and twice as likely to have psychosocial disabilities and physical and functional limitations as persons without cancer or chronic illness (Hewitt, Rowland, & Yancik, 2003). Compared with individuals under the age of 65 and without a history of cancer or other chronic illness, cancer survivors under the age of 65 are three times less likely to return to work. Not only can this have a devastating financial impact on the patient and family, but it can influence self-esteem and self-worth. Physical therapy provides helpful strategies to assist survivors in restoring their physical function and strength so that they can return to work.

Among the 14.5 million cancer survivors alive today in the United States, very few have had the opportunity to partake in a well-designed exercise program or have been referred to a physical therapy program for their impairments, he indicated. "We have a responsibility to improve the function of our patients as well as improve their cancer," Mr. Rizzo stated.

## INVOLVING PHYSICAL THERAPISTS IN CARE

Advanced practitioners in oncology are facing many challenges, many of which can be assisted by the role of the physical therapist and the rehabilitation team, he said. For example, the workforce is shrinking; more oncologists are retiring than are entering the profession. Oncologists and advanced practitioners (APs) have less quality time with patients and less time to spend on identifying treatment- or cancer-related impairments. Physical therapists who are involved in care early on may be able to fill in these gaps and may help define treatment goals that are often unclear in outpatient settings of fragmented care.

Treatment goals in physical therapy must be set through shared decision-making with the patient, continued Mr. Rizzo. "Commonly in medical treatments, patients are often passive recipients of care and unclear about their role in their disease management. In physical therapy, they become proactive participants in care, and are educated about their valuable role in maintaining optimal health and healthy lifestyle behaviors. "This helps with patient responsibility and accountability, and that in turn increases compliance in their medical treatments," he said.

## UNDERUTILIZATION OF PHYSICAL THERAPY

Exercise can play many roles for the cancer survivor. It has been shown to ameliorate physical and psychosocial side effects, improve cardiovascular, metabolic, and immune function, help restore proinflammatory/anti-inflammatory homeostasis, reduce health-care costs, and improve quality of life. There is also strong epidemiologic evidence that physical activity can improve survival, which has been eloquently shown in breast cancer (Holmes, Chen, Feskanich, Kroenke, & Colditz, 2005) and colorectal cancer (Meyerhardt et al., 2006).

Despite the many benefits of physical therapy, it is often underutilized in the oncology setting. There is often a lack of consensus as to when (or even if) to initiate an exercise program during treatment. Additionally, the use of drugs as first-line treatment "may marginalize the benefits of exercise," he added.

**Figure 1:**
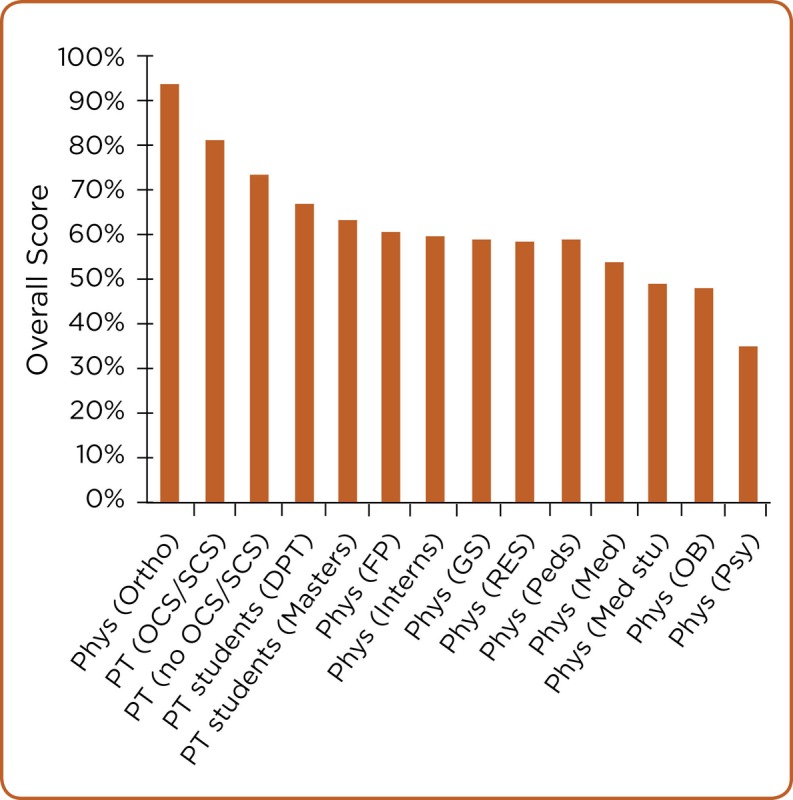
Musculoskeletal knowledge. Adapted with permission from Childs et al. (2005).

Many common impairments in patients with cancer are musculoskeletal—an area in which physical therapists are knowledgeable. One study compared the musculoskeletal knowledge of physical therapists with that of a large number of specialty physician groups, and only orthopedic surgeons scored higher than physical therapists (Childs et al., 2005; Figure 1).

## CANCER-RELATED FATIGUE

One area in which physical therapists can be useful is in managing cancer-related fatigue. Fatigue is experienced by up to 90% of patients treated with radiation therapy and up to 80% of those treated with chemotherapy; it may already be present in 40% of patients at diagnosis (Hofman, Ryan, Figueroa-Moseley, Jean-Pierre, & Morrow, 2007). The impact of fatigue on a patient’s quality of life, physical functioning, and ability to perform can be both profound and pervasive, explained Mr. Rizzo.

The National Comprehensive Cancer Center (NCCN) has developed guidelines for cancer-related fatigue, stating that exercise is the number one most effective nonpharmacologic intervention for this problem. The NCCN recommends physical therapy for patients with comorbidities, recent surgeries, functional and anatomic deficits, and substantial deconditioning.

## OSTEOPOROTIC PATIENTS

"Many physicians feel that their patients may be too frail to start an exercise program or will not make much progress in an exercise program, but studies have shown that the most deconditioned and frail [patients] make the greatest improvements in strength," he stated. In addition, he stressed the beneficial effects of postural and strength training, balance and endurance training, and pulmonary rehabilitation in the physical therapy setting for patients with osteoporosis, peripheral neuropathy, or other conditions that put patients at an increased risk of falling.

"It’s not just important to send them to physical therapy for muscle strengthening, but also to assess fracture risk and to appropriately identify their need for assistive devices for safety and efficiency," revealed Mr. Rizzo. The improper use of assistive devices can cause gait deviations and unnecessary energy expenditure, which can contribute to poor gait and fatigue.

## THE ROLE OF MYOKINES

Over the past decade, groundbreaking work in biochemistry and gene technology has promoted an understanding of the protective, healing, and regenerative role of myokines in cancer and of other chronic inflammatory conditions. Myokines are a type of cytokine released by muscles through muscle contraction, i.e., physical activity and exercise. Myokines can function in an autocrine, paracrine, and endocrine fashion, communicating with other organs like the brain, liver, and pancreas. Muscle contraction-induced myokines help maintain healthy tissue function, metabolism, immunomodulation, and embryogenesis throughout the body.

With high-intensity exercise, Interleukin-6 (IL-6) is a strong anti-inflammatory that can combat the pro-inflammatory side effects of chemotherapy; it has been shown to be released at 100-fold above resting muscle levels. This release is dose-dependent, associated with the mode, duration, and intensity of exercise (Pedersen, 2011). Improvements in muscle mass and muscle performance can directly attack proinflammatory cytokines, pointed out Mr. Rizzo.

Myostatin, a cytokine that restrains muscle proliferation and growth, has been shown to be released at higher levels in cachexic patients. During exercise, myokines such as brain-derived neurotrophic factor (BDNF) inhibit the release of myostatin, thus helping to slow the rate of muscle loss in advanced cancer patients.

**Figure 2:**
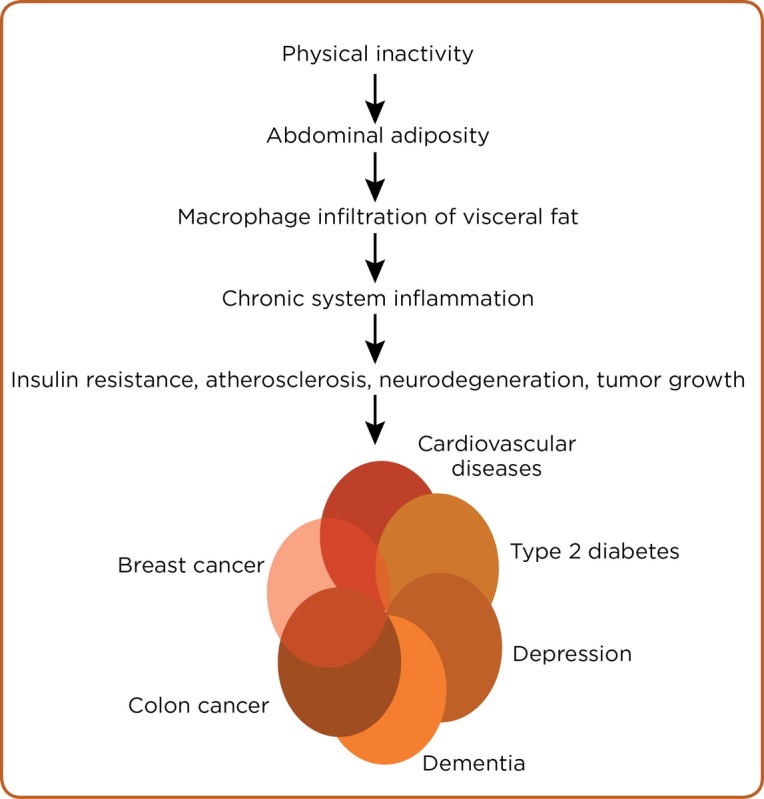
Inactivity leads to chronic inflammatory disease. Adapted from Pedersen (2011)

Physical inactivity leads to abdominal adiposity, which leads to macrophage infiltration of visceral fat, which then causes chronic systemic inflammation and insulin resistance, atherosclerosis, neurodegeneration, tumor growth, and a host of diseases (Figure 2). "Inflammation is the common denominator, and exercise can help by releasing powerful anti-inflammatory myokines," said Mr. Rizzo.

"Pharmaceutical companies are spending billions of dollars to try to replicate what muscle can do naturally," said Mr. Rizzo, who advocated a more conservative approach to prescribing drugs and a more liberal approach to using exercise as a component of treatment and self-management.

According to the American College of Sports Medicine Survivor Exercise Guidelines, it is never too early or too late to start an exercise program (Schmitz et al., 2010). The guidelines recommend 150 minutes/week of moderate-intensity or 75 minutes/week of high-intensity aerobic exercise, combined with resistance training and balance activities. Exercise is best accomplished by integrating it into the patient’s daily routine.

## RECAPPING THE PHYSICAL THERAPIST’S ROLE

According to Mr. Rizzo, advanced practitioners in oncology should identify and refer physical impairments early to physical therapy, incorporate physical therapists into the decision-making team, think conservatively before adding another drug to the treatment plan, and include the patient as a co-key decision-maker.

"To advance the patient’s quality of life and physical function, advanced practitioners in oncology should ask how to use the power of the muscular system for cell repair, defense, and healing against cancer treatment side effects and other inflammatory conditions," he concluded.
